# Microwave-Assisted Synthesis of New Selenazole Derivatives with Antiproliferative Activity

**DOI:** 10.3390/molecules18044679

**Published:** 2013-04-19

**Authors:** Adriana Ignat (Grozav), Luiza Gaina, Victor Kuete, Luminita Silaghi-Dumitrescu, Thomas Efferth, Valentin Zaharia

**Affiliations:** 1 Faculty of Pharmacy, “Iuliu Hatieganu” University of Medicine and Pharmacy, RO-400012, Victor Babes 41, Cluj-Napoca, Romania; E-Mail: vzaharia@umfcluj.ro; 2 Faculty of Chemistry and Chemical Engineering, “Babes Bolyai” University RO-400028, Arany Janos 11, Cluj-Napoca, Romania; E-Mail: lusi@chem.ubbcluj.ro; 3 Department of Pharmaceutical Biology, Institute of Pharmacy and Biochemistry, University of Mainz, Staudinger Weg 5, 55128 Mainz, Germany; E-Mails: kuetevictor@yahoo.fr (V.K.); efferth@uni-mainz.de (T.F.)

**Keywords:** selenazole, microwave assisted synthesis, cytotoxicity

## Abstract

New aryl-hydrazinyl-1,3-selenazole and aroyl-hydrazonyl-1,3-selenazoles were synthesized *via* Hantzsch type condensation reactions of selenosemicarbazides with α-halogenocarbonyl derivatives, under classical *versus* microwave heating conditions. Excellent yields and shorter reaction times were obtained under irradiation conditions. The structures of the synthesized compounds were assigned based on spectroscopic data (FT-IR, 1H-NMR), MS and elemental analysis. Selenazole derivatives were screened for their anti-proliferative effects against two leukemia cell lines (CCRF-CEM and HL60) and three carcinoma cell lines (MDA-MB231, HCT116 and U87MG).

## 1. Introduction

Selenium is an essential element for organisms, but its beneficial effects on human health are strongly dependent on concentration and on chemical form. The ingestion of foodstuffs with an elevated selenium content can induce toxicity [1], while a low concentration of Se leads to a deficient status [2].

In the last decade various organic selenium derivatives have been synthesized and tested for their biological properties such as free radical scavengers [3,4,5,6], superoxide anion-scavenging activity [7], inhibitors for different types of cancer cell proliferation [8,9,10,11,12,13], antioxidant activity [14,15,16] and anti-inflammatory activity or inhibitory effects on microglial activation [17]. Besides the beneficial effects, the toxic effects of selenium derivatives are well known; for example, the mutagenic effect induced by the sodium selenite in the yeast *Saccharomyces cerevisiae* is associated with the ability to act as an oxidizing agent producing superoxide and oxidative damage to DNA [18]. The genotoxic and cytotoxic effects of organoselonium derivatives in human leukocyte cells and V79 Chinese lung fibroblast cells can be associated to the pro-oxidant activity exhibited by selenium compounds when used in relatively high concentrations [19,20]. 

A literature survey reveals that the Hantzsch condensation (with changes in reaction conditions or catalysts) [21] appeared to be most frequently employed protocol for the synthesis of 1,3-selenazole derivatives. In our previous work, the lipophilicity and anticancer activity of a number of selenazole derivatives were investigated to better understand the correlation between drug delivery and permeation across a biological membrane [22,23,24]. Continuing our effort in the synthesis and in the characterization of the biological activity of new selenazole derivatives, we describe here the results of our investigations regarding the anti-proliferative effects against two leukemia cell lines and three carcinoma cell lines. To improve the reaction conditions, and encouraged by our previous results [25,26,27], microwave-assisted synthesis was used. The most important advantages induced by microwaves in organic synthesis consist in reduced reaction times, high yields and enhanced reaction rates [28].

## 2. Results and Discussion

**Scheme 1:**
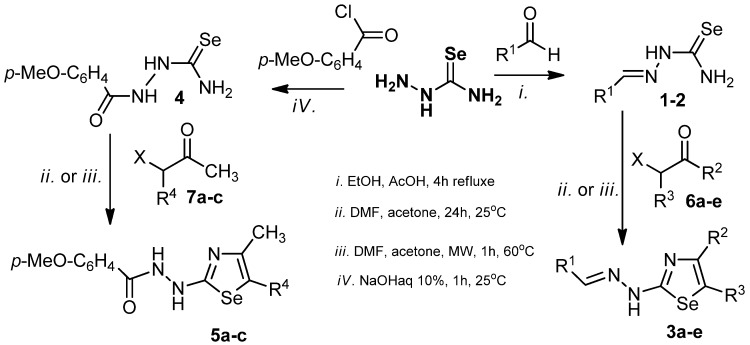
Synthesis of 1,3-selenazole derivatives.

The new arylidenehydrazinoselenazoles **3a**–**e** and aroylhydrazinoselenazoles **5a**–**c** were prepared *via* Hantzsch condensation reactions of a selenosemicarbazone or benzoylselenosemicarbazide with a series of α-halocarbonyl derivatives. Some of the starting materials, namely aryliden-selenosemicarbazones **1**–**2** and *p-*methoxybenzoylselenosemicarbazide 4, were obtained by the reaction of the corresponding aromatic carbaldehydes or methoxybenzoyl chloride with selenosemicarbazide (Scheme 1). Moderate yields were obtained using the classical reaction conditions, regardless of the various solvents tested (ethanol, acetone), and decomposition was observed when the reaction mixture was refluxed. To improve the yields and the reaction time, microwave irradiation was used instead of stirring the reaction mixture at room temperature. Experiments were therefore conducted at different temperatures (40 °C, 60 °C or 90 °C) and irradiation times (30', 60' and 90' respectively) to identify the optimal synthesis conditions. The best yields were obtained after 60 min of microwave irradiation at 60 °C inside the reaction vessel (temperatures above 90 °C cause decomposition of the reaction products).

**Table:** 

Comp.	R^1^	R^2^	R^3^	X	Comp.	R^1^	R^4^	X
**3a**	*p-*MeO-C_6_H_4_	COOEt	H		**1**	*p-*Cl-C_6_H_4_	-	-
**3b**	*p-*MeO-C_6_H_4_	Me	COMe		**2**	*p-*MeO-C_6_H_4_	-	-
**3c**	*p-*MeO-C_6_H_4_	CH_2_Cl	H		**5a**	-	COOEt	-
**3d**	*p-*Cl-C_6_H_4_	Me	COMe		**5b**	-	COMe	-
**3e**	*p-*Cl-C_6_H_4_	COOEt	H		**5c**	-	H	-
**6a**	-	COOEt	H	Br	**7a**	-	COOEt	Br
**6b**	-	Me	COMe	Cl	**7b**	-	COMe	Cl
**6c**	-	CH_2_Cl	H	Cl	**7c**	-	H	Cl
**6d**	-	Me	COMe	Cl				
**6e**	-	COOEt	H	Br				

A comparison of the two alternative techniques (see Table 1), microwave irradiation and the reaction at room temperature, emphasizes the advantages of microwave-assisted synthesis, which affords almost quantitative reaction yields in much shorter reaction times.

**Table 1:** Comparative yields in the synthesis of selenazole derivatives, under microwave irradiation and without heating.

Compounds	3a	3b	3c	3d	3e	5a	5b	5c
Yield (%) MW ^a^	93	92	95	94	91	94	93	87
Yield (%) ^b^	52	51	56	56	60	57	63	67

^a^ W irradiation, power P = 200 W, time 60 min; ^b^ Without heating, room temperature, time 1,440 min.

The structures of the newly-synthesized compounds were confirmed by their ^1^H-NMR, MS and FT-IR spectra. In the ^1^H-NMR spectra of selenazoles **3a**–**e** the most deshielded signal is a singlet around 8.1–8.9 ppm, assigned to the azomethine proton (CH=N). Other characteristic signals for compounds **3a**–**e** and **5a**–**c** are the two doublets generated by the aromatic protons from the phenyl ring. For example, these signals appear at 6.99 ppm, 7.66 ppm in the spectrum of selenazole **3a**, at 6.99 ppm, 7.68 ppm for selenazole **3b**, and 7.03 ppm, 7.95 ppm for selenazole **5a**.

The typical abundances for selenium isotopes ^76^Se(9.37%), ^77^Se(7.63%), ^78^Se(23.77%), ^80^Se(49.61%) and ^82^Se(8.73%) [29,30] are present in the EIMS spectra. The molecular ion, accompanied by the isotopic peaks confirms the molecular weight of the selenazole derivatives [*i.e.*, in the EIMS spectra for compound **5b**, all the isotopic peaks for selenium are present, 351(^78^Se), 350(^77^Se), 349(^76^Se), 353 (^80^Se), 355 (^82^Se)].

The synthesized compounds were screened for their anti-proliferative effects against two leukemia cell lines, CCRF-CEM and HL60, and three carcinoma cell lines MDA-MB231, HCT116 and U87MG, in a protocol employing the resazurin assay. The cut-off point for compounds exhibiting good cytotoxic properties is considered as 10 µM for the IC_50_ value following incubation between 48 and 72 h [31]. Values below this threshold were recorded with all studied compounds against the leukemia CCRF-CEM, showing that they could be useful in the development of anticancer compounds for this cell line. Additionally, compounds with the IC_50_ values closer to 10 µM (**3e** - 12.86 µM, **5c** - 11.94 µM and **4** - 10.62 µM) could also be suggested as useful cytotoxic compounds against leukaemia HL 60 cells. However, none of the compounds were as active as the reference drug doxorubicin. Although no significant anti-proliferative effects were noted against the three studied adherent cell lines, MDA-MB231, HCT116 and U87 MG, most of the compounds were active - with the IC_50_ values below 50 µM in the majority of the cases (Table 2).

**Table 2:** IC_50_ values of the newly-synthesized compounds, *versus* doxorubicin.

Compounds	Cell lines and IC_50_ values (µM)				
	CCRF-CEM	HL60	MDA-MB231	HCT116	U87MG
**3a**	6.36 ± 0.66	48.44 ± 11.14	>113.31	>113.31	>113.31
**3b**	8.87 ± 2.52	14.42 ± 234.31	72.60 ± 47.56	53.37 ± 8.67	66.53 ± 6.36
**3c**	5.11 ± 0.30	27.67 ± 8.45	85.24 ± 6.00	35.96 ± 4.17	65.41 ± 0.47
**3d**	9.97 ± 1.58	17.24 ± 1.66	42.68 ± 1.18	35.13 ± 3.77	30.32 ± 1.08
**3e**	8.40 ± 2.15	12.86 ± 1.99	65.72 ± 0.37	46.14 ± 0.97	59.12 ± 5.97
**4**	6.88 ± 1.53	10.62 ± 0.88	21.98 ± 0.63	23.51 ± 0.86	27.56 ± 10.02
**5a**	8.33 ± 2.03	29.88 ± 0.17	61.19 ± 4.86	24.99 ± 2.58	29.80 ± 1.68
**5b**	6.43 ± 0.96	13.23 ± 0.12	16.90 ± 4.55	22.25 ± 1.66	20.95 ± 1.62
**5c**	5.67 ± 3.87	11.94 ± 0.72	29.19 ± 1.92	34.66 ± 3.21	25.22 ± 7.23
**Doxorubicin**	0.20 ± 0.06	0.73 ± 0.20	1.10 ± 0.28	1.41 ± 0.29	1.06 ± 0.15

## 3. Experimental

### 3.1. General

All chemicals were obtained from standard commercial sources unless otherwise indicated. Compounds **1**, **2**, and **4** were prepared according to literature procedures [32,33]. Microwave irradiation was performed in a CEM Discover LabMate reactor. Melting points were measured with an Electrothermal IA 9200 apparatus, and are uncorrected values. ^1^H-NMR spectra were recorded in acetone-d_6_ (locked to Me_4_Si) using a 300 MHz or 400 MHz Bruker Avance NMR spectrometer. FT-IR spectra were recorded using a Bruker Vector 22 spectrometer. Elemental analysis was carried out on a Vario EL III instrument. The mass spectra were recorded on a Shimadzu QP 2010 Plus GC-MS instrument.

### 3.2. General Procedure for the Preparation of Arylhydrazinoselenazoles **3a**–**e**

(a) A solution of α-halocarbonyl derivative (2 mmol) in anhydrous acetone (5 mL) was added to a solution of benzylidenehydrazinecarboselenoamide derivative (2 mmol) in DMF (5 mL). The reaction mixture was stirred at room temperature for 24 h and then neutralized at pH = 7 with NaHCO_3_ aqueous solution (10%)._._ The precipitate was filtered and then recrystallized from ethanol.

(b) A solution of α-halocarbonyl derivative (2 mmol) in anhydrous acetone (5 mL) was added to a solution of benzylidenehydrazinecarboselenoamide derivative (2 mmol) in DMF (5 mL). The reaction mixture was introduced in a microwave reaction vessel, which was then sealed and subjected to microwave irradiation. The reaction mixture was subsequently neutralized at pH = 7 with NaHCO_3_ aqueous solution (10%). The obtained precipitate was filtered and then recrystallized from ethanol. Experimental parameters for all derivatives are listed in Table 1.

*(E)-Ethyl 2-[2-(4-methoxybenzylidene)hydrazinyl]-1,3-selenazole-4-carboxylate* (**3a**). White-yellowish crystals; yields: 36.7 mg, 52% (method a), 65.6 mg, 93% (method b); m.p. 216–217 °C; IR (KBr ν cm^−1^): 3181 (ν_NH_), 3113 (ν_CH_), 1714 (ν_COester_), 1612 (ν_C=N_), 1578, 1551, 1512 (ν_C=Caromatic_); EIMS (*m/z*): 355/353, 351/350, 349 (M^+^), 307, 134 (100%); ^1^H-NMR (300 MHz) δ: 1.31 (t, 3H, CH_3_, ^3^*J* = 7 Hz), 3.84 (s, 3H, OCH_3_), 4.26 (q, 2H, CH_2_, ^3^*J* = 7 Hz), 6.99 (d, 2H, ^3^*J* = 8.7 Hz), 7.66 (d, 2H, ^3^*J* = 8.7 Hz), 8.13 (s, 1H, CH), 8.18 (s, 1H, CH-Se); Anal. calcd. for C_14_H_15_N_3_O_3_Se: C 47.74, H 4.29, N 11.93, O 13.63, Se, 22.42; found: C 47.76, H 4.25, N 11.98, O 13.54.

*(E)-1-[2-(2-(4-Methoxybenzylidene)hydrazinyl)-4-methyl-1,3-selenazol-5-yl]ethanone* (**3b**). Brown crystals; yields: 34.3 mg, 51% (method a), 62 mg, 92% (method b); m.p. 209–210 °C; IR (KBr ν cm^−1^): 3184 (ν_NH_), 3030 (ν_CH_), 1699 (ν_COcetone_), 1614 (ν_C=N_), 1575, 1550, 1511 (ν_C=Caromatic_); EIMS (*m/z*): 339/337, 335/334, 333 (M^+^), 294 (100%); ^1^H-NMR (300 MHz) δ: 2.40 (s, 3H, CH_3_), 2.49 (s, 3H, CH_3_), 3.84 (s, 3H, CH_3_), 6.99 (d, 2H, ^3^*J* = 8.7 Hz), 7.68 (d, 2H, ^3^*J* = 8.7 Hz), 8.19 (s, 1H, CH=N); Anal. calcd for C_14_H_15_N_3_O_2_Se: C 50.01, H 4.50, N 12.50, O 9.52, Se 23.48; found: C 50.04, H 4.52, N 12.51, O 9.46.

*(E)-4-(Chloromethyl)-2-[2-(4-methoxybenzylidene)hydrazinyl]-1,3-selenazole* (**3c**). White crystals; yields: 36.7 mg, 56% (method a), 62.4 mg, 95% (method b); m.p. 183–184 °C; IR (KBr ν cm^−1^): 3182 (ν_NH_), 3058 (ν_CH_), 1610 (ν_C=N_), 1576, 1551, 1510 (ν_C=Caromatic_); EIMS (*m/z*):331/329, 327/326, 325 (M^+^), 294, 134 (100%); ^1^H-NMR (300 MHz) δ: 3.83 (s, 3H, CH_3_), 4.5 (s, 2H, CH_2_Cl), 6.98 (d, 2H, ^3^J = 8.7 Hz), 7.27 (s, 1H, Se-CH), 7.64 (d, 2H, ^3^J = 8.7 Hz), 8.10 (s, 1H, CH=N); Anal. calcd for C_12_H_12_ClN_3_OSe: C 43.85, H 3.68, Cl 10.79, N 12.79, O 4.87, Se 24.03; found: C 43.87, H 3.69, N 12.81, O 4.82.

*(E)-1-[2-(2-(4-Chlorobenzylidene)hydrazinyl)-4-methyl-1,3-selenazol-5-yl]ethanone* (**3d**). Brown crystals; yields: 38.1 mg, 56% (method a), 64 mg, 94% (method b); m.p 179–180 °C; IR (KBr ν cm^−1^): 3184 (ν_NH_), 2993 (ν_CH_), 1703 (ν_CO cetone_), 1613 (ν_C=N_), 1577, 1551, 1511 (ν_C=Caromatic_); EIMS (*m/z*): 343/341, 339/338, 337 (M^+^), 298, 188, 138 (100); ^1^H-NMR (300 MHz) δ: 2.41 (s, 3H, CH_3_), 2.5 (s, 3H, CH_3_), 7.42 (d, 2H, ^3^*J* = 7.3 Hz), 7.7(d, 2H, ^3^*J* = 7.3 Hz), 8.14 (s, 1H, CH=N). Anal. calcd for C_13_H_12_ClN_3_OSe: C 45.83, H 3.55, Cl, 10.41, N 12.33, O 4.70, Se, 23.18; found: C 45.86, H 3.58, N 12.35, O 4.62.

*(E)-Ethyl 2-[2-(4-chlorobenzylidene)hydrazinyl]-1,3-selenazole-4-carboxylate* (**3e**). White-yellowish crystals; yields: 42.8 mg, 60% (method a), 64.9 mg 91% (method b); m.p. 240–241 °C; IR (KBr ν cm^−1^): 3182 (ν_NH_), 3002 (ν_CH_), 1722 (ν_COester_), 1614 (ν_C=N_), 1576, 1550, 1512 (ν_C=Caromatic_); EIMS (*m/z*): 359/357(100%), 355/354, 325 (M^+^), 311, 246, 138, 111; ^1^H-NMR (300 MHz) δ : 1.24 (t, 3H, CH_3_, ^3^*J* = 7 Hz), 4.16 (q, 2H, CH_2_, ^3^*J* = 7 Hz), 7.53 ppm (d, 2H, ^3^*J* = 7.6 Hz), 7.97 (d, 2H, ^3^*J* = 7.6 Hz), 8.13 (s, 1H, CH), 8.33 (s, 1H, CH-Se). Anal. calcd for C_13_H_12_ClN_3_O_2_Se: C 43.78, H 3.39, Cl, 9.94; N 11.78, O 8.97, Se, 22.14; found: C 43.80, H 3.41, N 11.79, O 8.93. 

### 3.3. General Procedure for the Preparation of Aroylhydrazinoselenazoles **5a**–**c**

(a) A solution of α-halogenocarbonyl derivative (2 mmol) in anhydrous acetone (5 mL) was added to a solution of 2-(4-methoxybenzoyl)hydrazinecarboselenoamide **4** (54.7mg, 2 mmol) in DMF (5 mL). The reaction mixture was stirred at room temperature for 24 h and then neutralized at pH = 7 with NaHCO_3_ aqueous solution (10%)_._ The precipitate was filtered and then recrystallized from ethanol.

(b) A solution of α-halogenocarbonyl derivative (2 mmol) in anhydrous acetone (5 mL) was added to a solution of 2-(4-methoxybenzoyl)hydrazinecarboselenoamide **4** (54.7mg, 2 mmol) in DMF (5 mL). The reaction mixture was introduced in a microwave reaction vessel, which was then sealed and subjected to microvawe irradiation. The reaction mixture was subsequently neutralized at pH = 7 with NaHCO_3_ aqueous solution (10%)_._ The obtained precipitate was filtered and then recrystallized from ethanol. Experimental parameters for all derivatives are listed in Table 1.

*Ethyl 2-[2-(4-methoxybenzoyl)hydrazinyl]-4-methyl-1,3-selenazole-5-carboxylate* (**5a**). White crystals; yields: 43.6 mg, 57% (method a), 72 mg, 94% (method b); m.p. 177–178 °C; IR (KBr ν cm^−1^): 3412, 3176 (ν_NH_), 2982, 2838 (ν_CH_), 1700 (ν_COester_), 1631 (ν_COamide_), 1587, 1531 (ν_C=Caromatic_); EIMS (*m/z*): 385/383, 381/380, 379 (M^+^), 135 (100%); ^1^H-NMR (400 MHz) δ: 1.23 (t, 3H, CH_3_, ^3^*J* = 7 Hz), 2.43 (s, 3H, CH_3_), 3.84 (s, 3H, CH_3_), 4.16 (q, 2H, CH_2_, ^3^*J* = 7 Hz), 7.03 ppm (d, 2H, ^3^*J* = 8.2 Hz), 7.95 (d, 2H, ^3^*J* = 8.2 Hz); Anal. calcd for C_15_H_17_N_3_O_4_Se: C 47.13, H 4.48, N 10.99, O 16.74, Se, 20.66; found: C 47.15, H 4.50, N 11.01, O 16.23.

*N'-(5-Acetyl-4-methyl-1,3-selenazol-2-yl)-4-methoxybenzohydrazide* (**5b**). White crystals; yields: 44 mg, 63% (method a), 65.6 mg, 93% (method b); m.p. 186–187 °C; IR (KBr ν cm^−1^): 3412, 3211 (ν_NH_), 3097, 2946 (ν_CH_), 1696 (ν_CO cetone_), 1635 (ν_CO amide_), 1596, 1532 (ν_C=Caromatic_); EIMS (*m/z*): 355/353, 351/350, 349 (M^+^), 135 (100%); ^1^H-NMR (300 MHz) δ: 2.4 (s, 3H, CH_3_), 2.46 (s, 3H, CH_3_), 3.89 (s, 3H, CH_3_), 7.05 (d, 2H, ^3^*J* = 8.5 Hz), 7.96 (d, 2H, ^3^*J* = 8.5 Hz); Anal. calcd. for C_14_H_15_N_3_O_3_Se: C 47.74, H 4.29, N 11.93, O 13.63, Se 22.42; found: C 47.76, H 4.31, N 11.95, O 13.54.

*4-Methoxy-N'-(4-methyl-1,3-selenazol-2-yl)benzohydrazide* (**5c**). White crystals; yields: 41.6 mg, 67% (method a), 54.1 mg 87% (method b); m.p. 265–266 °C; IR (KBr ν cm^−1^): 3399, 3208 (ν_NH_), 3093, 2944 (ν_CH_), 1633 (ν_CO amidă_), 1591, 1526 (ν_C=Caromatic_); EIMS (*m/z*): 313/311, 309/308, 307 (M+), 135 (100%). ^1^H-NMR (400 MHz); δ: 2.4 (s, 3H, CH_3_), 3.8 (s, 3H, CH_3_), 7.05 (d, 2H, ^3^*J* = 8.5 Hz), 7.12(s, 1H, Se-CH), 7.95 (d, 2H, ^3^*J* = 8.5 Hz); Anal. calcd. for C_12_H_13_N_3_O_2_Se: C 46.46, H 4.22, N 13.55, O 10.32, Se 25.45; found: C 46.43, H 4.25, N 13.53, O 10.30.

### 3.4. Cytotoxicity Assay

The resazurin reduction assay [34] was performed to assess the cytotoxicity of the newly-synthesized compounds towards various sensitive and resistant cancer cell lines, including the leukemia CCRF-CEM, HL60, breast MDA-MB231, colon HCT116 and glioblastoma U87MG. The assay is based on the reduction of the indicator dye, resazurin, to the highly fluorescent resorufin by viable cells. Non-viable cells rapidly lose their metabolic capacity to reduce resazurin and, thus, do not produce fluorescent signals anymore. Briefly, adherent cells were detached by treatment with 0.25% trypsin/EDTA (Invitrogen, Darmstadt Germany) and an aliquot of 1 × 10^4^ cells was placed in each well of a 96-well cell culture plate (Thermo Scientific, Langenselbold, Germany) in a total volume of 200 µL. Cells were allowed to attach overnight and then were treated with different concentrations of compounds. For suspension cells, aliquots of 2 × 10^4^ cells per well were seeded in 96-well-plates in a total volume of 100 µL. The studied compound was immediately added in varying concentrations in an additional 100 µL of culture medium to obtain a total volume of 200 µL/well. After 72 h, resazurin (Sigma-Aldrich, Schnelldorf, Germany) (20 µL, 0.01% w/v) in ddH_2_O was added to each well and the plates were incubated at 37 °C for 4 h. Fluorescence was measured on an Infinite M2000 Pro^TM^ plate reader (Tecan, Crailsheim, Germany) using an excitation wavelength of 544 nm and an emission wavelength of 590 nm. Each assay was done at least twice with six replicates each. The viability was evaluated based on a comparison with untreated cells. IC_50_ values represent the compound concentrations required to inhibit 50% of cell proliferation and were calculated from a calibration curve by linear regression using Microsoft Excel.

## 4. Conclusions

In conclusion, we have carried out, in high yield and short reaction times, the synthesis of eight new 1,3-selenazole derivatives by microwave irradiation. The new 1,3-selenazoles were investigated for anti-proliferative effects against two leukemia cell lines (CCRF-CEM and HL60) and three carcinoma cell lines (MDA-MB231, HCT116 and U87MG) and show moderate biological *in vitro* activity. 

## References

[1] Muller D., Desel H. (2010). Acute selenium poisoning by paradise nuts (Lecythis ollaria). Hum. Exp. Toxicol..

[2] Klapec T., Mandić M.L., Grgić J., Primorac L., Ikić M., Lovrić T., Grgić Z., Herceg Z. (1998). Daily dietary intake of selenium in eastern Croatia. Sci. Total Environ..

[3] Nishina A., Kimura H., Kozawa K., Sommen G.L., Nakamura T., Heimgartner H., Koketsu M., Furukawa S. (2011). A superoxide anion-scavenger, 1,3-selenazolidin-4-one suppresses serum deprivation-induced apoptosis in PC12 cells by activating MAP kinase. Toxicol. Appl. Pharmacol..

[4] Sekiguchi A., Nishina A., Kimura H., Fukumoto R., Kogami M., Ishihara H., Koketsu M. (2006). Bis-(2-amino-5-selenazoyl) ketone as a superoxide anion-scavenger. Pharm. Bull..

[5] Takahashi H., Nishina A., Kimura H., Motoki K., Koketsu M., Ishihara H. (2004). Tertiary selenoamide compounds are useful superoxide radical scavengers *in vitro*. Eur. J. Pharm. Sci..

[6] Takahashi H., Nishina A., Fukumoto R., Kimura H., Koketsu M., Ishihara H. (2005). Selenoureas and thioureas are effective superoxide radical scavengers *in vitro*. Life Sci..

[7] Takahashi H., Nishina A., Fukumoto R., Kimura H., Koketsu M., Ishihara H. (2005). Selenocarbamates are effective superoxide anion scavengers *in vitro*. Eur. J. Pharm. Sci..

[8] Bijian K., Zhang Z., Xu B., Jie S., Chen B., Wan S., Wu J., Jiang T., Alaoui-Jamali M.A. (2012). Synthesis and biological activity of novel organoselenium derivatives targeting multiple kinases and capable of inhibiting cancer progression to metastases. Eur. J. Med. Chem..

[9] Madhunapantula S.V., Desai D., Sharma A., Huh S.J., Amin S., Robertson G.P. (2008). PBISe, a novel selenium-containing drug for the treatment of malignant melanoma. Mol. Cancer Ther..

[10] Desai D., Madhunapantula S.V., Gowdahalli K., Sharma A., Chandagaludoreswamy R., El-Bayoumy K., Robertson G.P., Amin S. (2010). Synthesis and characterization of a novel iNOS/Akt inhibitor Se,Se0-1,4-phenylenebis(1,2-ethanediyl)bisisoselenourea (PBISe)—against colon cancer. Bioorg. Med. Chem. Lett..

[11] Xing F., Li S., Ge X., Wang C., Zeng H., Li D., Dong L. (2008). The inhibitory effect of a novel organoselenium compound BBSKE on the tongue cancer Tca8113 *in vitro* and *in vivo*. Oral Oncol..

[12] Wang L., Yang Z., Fu J., Yin H., Xiong K., Tan Q., Jin H., Li J., Wang T., Tang W. (2012). Ethaselen: a potent mammalian thioredoxin reductase 1 inhibitor and novel organoselenium anticancer agent. Free Radic. Biol. Med..

[13] Terazawa R., Garud D.R., Hamada N., Fujita Y., Itoh T., Nozawa Y., Nakane K., Deguchi T., Koketsu M., Ito M. (2010). Identification of organoselenium compounds that possess chemopreventive properties in human prostate cancer LNCaP cells. Bioorg. Med. Chem..

[14] Merino-Montiel P., Maza S., Martos S., LOpez O., Maya I., Fernandez-Bolanos J.G. (2013). Synthesis and antioxidant activity of *O*-alkyl selenocarbamates, selenoureas and selenohydantoins. Eur. J. Pharm. Sci..

[15] Barbieri Gerzson M.F., Victoria F.N., Radatz C.S., de Gomes M.G., Boeira S.P., Jacob R.G., Alves D., Jesse C.R., Savegnago L. (2012). *In vitro* antioxidant activity and *in vivo* antidepressant-like effect of α-(phenylselanyl) acetophenone in mice. Pharmacol. Biochem. Behav..

[16] Acker C.I., Brandão R., Rodrigues Rosário A., Nogueira C.W. (2009). Antioxidant effect of alkynylselenoalcohol compounds on liver and brain of rats *in vitro*. Environ. Toxicol. Phar..

[17] Nam K.N., Koketsu M., Lee E.H. (2008). 5-Chloroacetyl-2-amino-1,3-selenazoles attenuate microglial inflammatory responses through NF-κB inhibition. Eur. J. Pharmacol..

[18] Letavayova L., Vlasakova D., Spallholz J.E., Brozmanova  J., Chovanec M. (2008). Toxicity and mutagenicity of selenium compounds in Saccharomyces cerevisiae. Mutat. Res..

[19] Santos D.B., Schiar V.P.P., Ribeiro M.C.P., Schwab R.S., Meinerz D.F., Allebrandt J., Rocha J.B.T., Nogueira C.W., Aschner M., Barbosa N.B.V. (2009). Genotoxicity of organoselenium compounds in human leukocytes *in vitro*. Mutat. Res..

[20] Rosa R.M., Picada J.N., Saffi J., Henriques J.A.P. (2007). Cytotoxic, genotoxic, and mutagenic effects of diphenyl diselenide in Chinese hamster lung fibroblasts. Mutat. Res..

[21] Tao H., Weng Y., Zhuo Z., Chang G., Urbatsch L.I., Zhang Q. (2011). Design and synthesis of Selenazole-containing peptides for cocrystallization with P-glycoprotein. ChemBioChem.

[22] Cozma A., Vlase L., Ignat A., Zaharia V., Gocan S., Marutoiu C., Fodor A. (2012). Lipophilicity study of new selenazole derivatives by RP-HPLC. Rev. Chim. (Bucharest).

[23] Cozma A., Zaharia V., Ignat A., Gocan S., Grinberg N. (2012). Prediction of the lipophilicity of nine new synthesized selenazoly and three aroyl–hydrazinoselenazoles derivatives by reversed-phase high performance thin-layer chromatography. J. Chromatogr. Sci..

[24] Zaharia V., Ignat A., Ngameni B., Kuete V., Moungang M.L., Fokunang C.N., Vasilescu M., Palibroda N., Cristea C., Silaghi-Dumitrescu L. (2013). Heterocycles 23: Synthesis, characterization and anticancer activity of new hydrazinoselenazole derivatives. Med. Chem. Res..

[25] Gaina L., Gal E., Mataranga-Popa L., Porumb D., Nicolescu A., Cristea C., Silaghi-Dumitrescu L. (2011). Synthesis, structural investigations, and DFT calculations on novel 3-(1,3-dioxan-2-yl)-10-methyl-10*H*-phenothiazine derivatives with fluorescence properties. Tetrahedron.

[26] Gaina L., Porumb D., Silaghi-Dumitrescu L., Cristea C., Silaghi-Dumitrescu L. (2010). On the microwave-assisted synthesis of acylphenothiazine derivatives-Experiment versus theory synergism. Can. J. Chem..

[27] Gaina L., Cristea C., Moldovan C., Porumb D., Surducan E., Deleanu C., Mahamoud A., Barbe J., Silberg I.A. (2007). Microwave-assisted synthesis of phenothiazine and quinoline derivatives. Int. J. Mol. Sci..

[28] Vaibhav P.M., Van der Eycken E.V. (2011). Microwave-assisted C-C bond forming cross-coupling reactions: an overview. Chem. Soc. Rev..

[29] Audi G., Wapstra A.H. (1993). The 1993 atomic mass evaluation. Nucl. Phys. A.

[30] Audi G., Wapstra A.H. (1995). The 1995 update to the atomic mass evaluation. Nucl. Phys. A.

[31] Brahemi G., Kona F.R., Fiasella A., Buac D., Soukupov J., Brancale A., Burger A.M., Westwell A.D. (2010). Exploring the structural requirements for inhibition of the ubiquitin E3 ligase breast cancer associated protein 2 (BCA2) as a treatment for breast cancer. J. Med. Chem..

[32] Wiles D.M., Suprunchuk T. (1967). The C==S stretching vibration in the infrared spectra of some thiosemicarbazones. II. Aldehyde thiosemicarbazones containing aromatic groups. Can. J. Chem..

[33] Collard-Charon C., Renson M. (1962). Synthèse des Sélénosemicarbazides Substituées III. Synthèse des sélénosemicarbazides substituées en 1. Bull. Soc. Chim. Belg..

[34] O’Brien J., Wilson I., Orton T., Pognan F. (2000). Investigation of the Alamar Blue (resazurin) fluorescent dye for the assessment of mammalian cell cytotoxicity. Eur. J. Biochem..

